# GPS-Lipid: a robust tool for the prediction of multiple lipid modification sites

**DOI:** 10.1038/srep28249

**Published:** 2016-06-16

**Authors:** Yubin Xie, Yueyuan Zheng, Hongyu Li, Xiaotong Luo, Zhihao He, Shuo Cao, Yi Shi, Qi Zhao, Yu Xue, Zhixiang Zuo, Jian Ren

**Affiliations:** 1 State Key Laboratory of Biocontrol, School of Life Sciences, Sun Yat-sen University, Guangzhou, Guangdong 510275, China; 2 Sun Yat-sen University Cancer Center, State Key Laboratory of Oncology in South China, Collaborative Innovation Center for Cancer Medicine, Guangzhou 510060, China; 3 Collaborative Innovation Center of High Performance Computing, National University of Defense Technology, Changsha 410073, China; 4 Department of Biomedical Engineering, College of Life Science and Technology, Huazhong University of Science and Technology, Wuhan 430074, China

## Abstract

As one of the most common post-translational modifications in eukaryotic cells, lipid modification is an important mechanism for the regulation of variety aspects of protein function. Over the last decades, three classes of lipid modifications have been increasingly studied. The co-regulation of these different lipid modifications is beginning to be noticed. However, due to the lack of integrated bioinformatics resources, the studies of co-regulatory mechanisms are still very limited. In this work, we developed a tool called GPS-Lipid for the prediction of four classes of lipid modifications by integrating the Particle Swarm Optimization with an aging leader and challengers (ALC-PSO) algorithm. GPS-Lipid was proven to be evidently superior to other similar tools. To facilitate the research of lipid modification, we hosted a publicly available web server at http://lipid.biocuckoo.org with not only the implementation of GPS-Lipid, but also an integrative database and visualization tool. We performed a systematic analysis of the co-regulatory mechanism between different lipid modifications with GPS-Lipid. The results demonstrated that the proximal dual-lipid modifications among palmitoylation, myristoylation and prenylation are key mechanism for regulating various protein functions. In conclusion, GPS-lipid is expected to serve as useful resource for the research on lipid modifications, especially on their co-regulation.

Most genes in eukaryotic cell are post-translationally modified by a wide range of chemical groups. Among those post-translational modifications, the addition and removal of lipid groups to certain amino acids is a key modification that orchestrates the subcellular trafficking^1^,^2^, signaling^3^,^4^ and membrane association^5^ of proteins. With the rapid development of numerous innovative techniques, three prevalent forms of lipid modifications, such as S-palmitoylation, prenylation and N-myristoylation, are now extensively studied.

The reversible attachment of a 16-carbon fatty acid palmitate to protein via thioester linkage is called as S-palmitoylation^6^. By effectively increasing the hydrophobicity of its modified substrates, the S-palmitoylation process can dynamically regulate the membrane association of various cellular proteins^1^,^2^. Cellular proteins may also be covalently modified with the 14-carbon saturated fatty acid myristate, which is known as N-myristoylation. By recognizing a MGXXXS/T signature at N-terminus, the N-myristoyl transferase (NMT) may catalyze the addition of myristate to glycine via an amide bond^7^,^8^. Another important type of lipid modification is prenylation. This process involves the addition of a 15-carbon farnesyl group or a 20-carbon geranylgeranyl group to a C-terminal cysteine that conform to a consensus CAAX motif 9. Typically, the farnesylation is catalyzed by protein farnesyltransferase (FTase)^10^, whereas the geranylgeranylation is performed by protein geranylgeranyltransferase type I (GGTase-I)^11^,^12^. However, in case of Rab proteins, the geranylgeranyltransferase type II (GGTase-II) which recognized a C-terminal CC/CXC motif is found to catalyze the geranylgeranylation process^9^,^13^. Although the enzymatic processes of protein lipidation vary greatly, different types of lipid groups are still found to modify similar protein substrates, which implies a strong co-regulation between different lipid modifications. One of the most striking example is the regulation of small GTPases in subcellular trafficking by prenylation and palmitoylation^14^. In Ras and Rho families, palmitoylation frequently occurs in the hypervariable domain that adjacents to the prenylated C-terminal end CAAX box^15^. These two types of lipid modification provide sufficient hydrophobicity for proteins to localize on cellular membranes and the precise subcellular localizations of these small GTPases are essential for their proper functionalities^16^. Additionally, with the help of an intracellular palmitoylation–depalmitoylation cycle, the prenylated small GTPases are able to dynamically traffic from Golgi apparatus to plasma membranes^17^. A similar co-regulatory mechanism was also reported between myristoylation and palmitoylation. Given the fact that the myristoylation, by itself, is not providing enough hydrophobicity of the modified protein for its membrane association^18^, extra N-terminal palmitoylation on the myristoylated proteins are usually required for the stable membrane attachment and translocation to rafts/caveolae or intracellular liquid-ordered domains^19^,^20^. Some Guanylate Cyclase Activating Proteins^21^, most of the members of the Src family of protein tyrosine kinases^22^ and the Giα subfamily of alpha subunits of G proteins^23^ are examples that undergo this kind of regulation. Thus, dual-lipid modifications are responsible for the correct localization of many signaling proteins, and play crucial roles in coordinating the extracellular stimuli and intracellular signaling.

Due to their essential physiological functions, the dysfunctions of lipid modification may lead to many sorts of diseases. For example, the overexpression of palmitoyl acyltransferases (PATs) may implicate in schizophrenia^24^ and Huntington’s disease^25^. The N-myristoylation is observed to mediate the viral Infectivity and eukaryotic infections^26^. Taken together, the research on lipid modification, especially on the co-regulatory mechanisms, will be particularly important for identifying potential drug targets for further diagnostic and therapeutic consideration. However, due to the limitations of integrative bioinformatics resources, the overall investigations that focusing on the co-regulation of lipid modifications are seldom performed. This deficiencies may grievously hamper the development of effective therapies for disorders related to lipid modifications.

Recently, several prediction tools for lipid modifications were constructed. CSS-Palm^27^ and CKSAAP-Palm^28^ are two widely-used tools that can be used to predict palmitoylation sites. NMT^7^ and Myristoylator^29^ were specifically designed for N-myristoylation prediction. PrePS^9^ was proposed to predict protein CAAX farnesylation, CAAX geranylgeranylation and Rab geranylgeranylation sites. Unfortunately, none of them can predict a complete set of lipid modification sites and assist the research on co-regulatory mechanisms between different lipid modifications. Beyond that, the number of experimentally identified lipidation sites has been significantly expanded in recent years, which provides an opportunity for improving the performance of lipid modification sites prediction.

In this work, we present GPS-Lipid, which is a comprehensive predictor for multiple protein lipid modification sites. From the published literatures, we manually collected 737 S-palmitoylation sites in 361 proteins, 106 S-farnesylation sites in 97 proteins, 95 S- geranylgeranylation sites in 70 proteins and 283 N-myristoylation sites in 281 proteins. Using this comprehensive dataset, we developed a new algorithm called GPS-Lipid, in which we employed the ALC-PSO^30^ method in our previous GPS algorithm for model training . Both a standalone package and an online service were freely available at http://lipid.biocuckoo.org.

## Results

### Development of GPS-Lipid for the prediction of lipid modification sites

Previously, we developed GPS (Group-based Prediction System) algorithm and successfully applied it to the prediction of post-translational modification sites such as phosphorylation^31^ and sumoylation^32^. In this work, for the prediction of protein lipid modification sites, we developed an update version of GPS algorithm, which adopted the ALC-PSO strategy as shown in Fig. S1 to prevent premature convergence and maintain fast training feature. We named this updated tool as GPS-Lipid.

**Figure 1:**
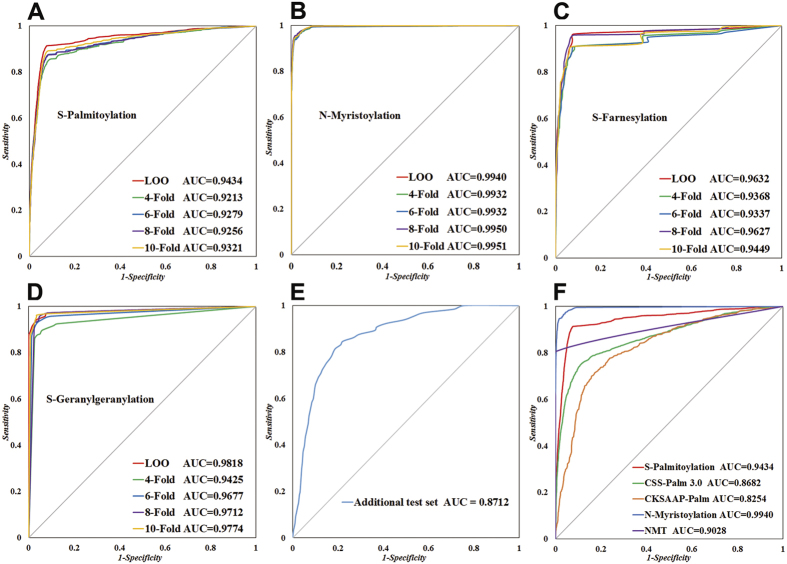
Performance evaluation and comparison of GPS-Lipid. (**A–D**) The performance evaluation for the predictions of S-palmitoylation, N-myristoylation, S-farnesylation and S-geranylgeranylation. The LOO and 4-,6-,8-,10-fold cross validation were performed. (**E**) An additional test set that was not included in the training set was applied to carry out the further evaluation of palmitoylation prediction. (**F**) The performance comparison among GPS-Lipid, CSS-Palm, CKSAAP-Palm and NMT. To avoid any bias, the same data set was used and the LOO validation was performed.

To evaluate the performance of GPS-Lipid, LOO and 4-, 6-, 8-, 10-fold cross validation were performed for S-palmitoylation, N-myristoylation, S-farnesylation and S-geranylgeranylation, respectively. Apparently, the AUCs for all tests are larger than 0.9 (Fig. 1A–D), indicating that GPS-Lipid is an accurate predictor. Furthermore, the ROC curves of 4-, 6-, 8-, 10-fold cross validations were found to be close to LOO validation in all lipid modification predictors, which demonstrates that GPS-Lipid is also a robust predictor. Since the positive and negative dataset are highly imbalanced in our training set, we calculated the precision-recall curves to further evaluate the performance of GPS-Lipid. A similar result was observed, which further confirmed that GPS-Lipid is an accurate and robust predictor even in the case of imbalanced training set (Fig. S2). To further evaluate the accuracy of GPS-Lipid, an independent test dataset was used. Due to data size limitation, only S-palmitoylation were evaluated. The AUC of the prediction for this independent test dataset was 0.8712 (Fig. 1E), further indicating the robustness of GPS-Lipid.

We then compared GPS-Lipid with known predictors such as NMT, CSS-Palm and CKSAAP-Palm. The LOO validation was carried out for all predictors. As a result, the performance of GPS-Lipid is superior to all other predictors: the AUCs for palmitoylation and myristoylation prediction using GPS-Lipid are 0.9434 and 0.9940, respectively, while the AUCs for CSS-Palm, CKSAAP-Palm and NMT are 0.8682, 0.8254 and 0.9028, respectively (Fig. 1F).

### Availability and Utility of GPS-Lipid

**Figure 2:**
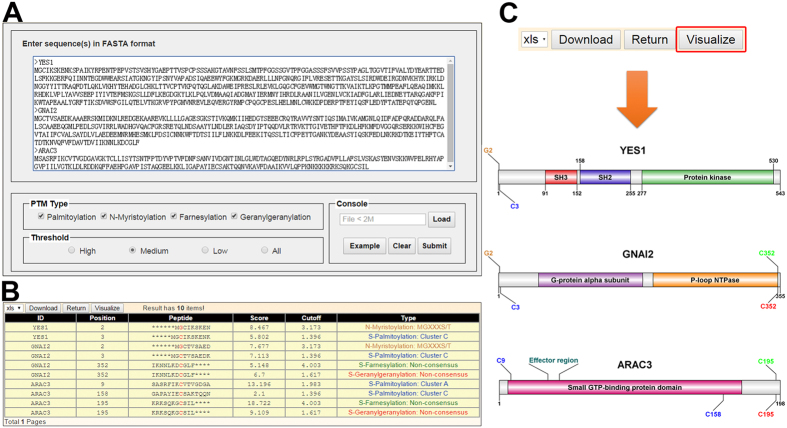
A snapshot of GPS-Lipid. (**A**) The human tyrosine-protein kinase Yes (YES1), mouse guanine nucleotide-binding protein G(i) subunit alpha-2 (GNAI2) and *Arabidopsis thaliana* rac-like GTP-binding protein (ARAC3) were taken as an example to try out the predictor. All the four supported lipidation were selected and predicted using the default threshold. (**B**) The predicted results of these three protein sequences. Different modification types were marked with different colors. (**C**) Visualization of the predicted results. By clicking on the “visualize” button in the result page, the lipid modification sites are illustrated in a domain graph. To distinguish between different lipid modifications, the visualization tool will marked them with different colors.

GPS-Lipid can be easily accessed through either standalone package or interactive web server. The GPS-Lipid web server provides a friendly interactive interface (Fig. 2A). Below the “sequence panel”, many interactive options are provided to help users configure their own run of GPS-Lipid. The “PTM Type panel” lists four supported lipid modification types to allow users conveniently predict any combinations of lipid modifications. The “threshold panel” provides three thresholds to allow users run GPS-Lipid with high, medium and low stringency. The “console panel” allows users to input the sequences through file and provides protein sequences example to run GPS-Lipid.

To clarify the utility of GPS-Lipid web server in more detail, here we took the protein sequences of human tyrosine-protein kinase Yes (YES1), mouse guanine nucleotide-binding protein G(i) subunit alpha-2 (GNAI2) and *Arabidopsis thaliana* rac-like GTP-binding protein (ARAC3) as an example. Multiple sequences can be inputted for GPS-Lipid via FASTA format. Alternatively, the sequences could also be uploaded as a FASTA format file. Using a default threshold, the potential lipid modification sites of our example are predicted and presented as shown in Fig. 2B. YES1 is known to be modified by N-myristoyl group at N-terminal glycine, which is essential for its regulation role in cellular transformation^33^. As expected, GPS-Lipid successfully predicted the N-myristoylation at N-terminal glycine. As for GNAI2, GPS-Lipid identified an N-myristoylation site at position 2, which has been reported to associate with the constitutive activation of alpha i2 signal transduction functions^34^. We also identified several geranylgeranylation sites in the C-terminal of ARAC3 where a CAAX motif has been identified through an in vitro prenylation screen^35^,^36^. Taken together, GPS-Lipid is able to identify different kinds of known lipid modification sites, indicating its robustness and reliability.

In addition to the lipid modification predictor, we also developed a visualization tool to generate a schematic diagram as shown in Fig. 2C. By marking the functional domains with their precise lipid modification sites in proteins, the visualization tool can assist researchers in illustrating the underlying mechanisms of lipid modification process.

### The co-regulatory mechanisms in Lipid modifications

**Figure 3:**
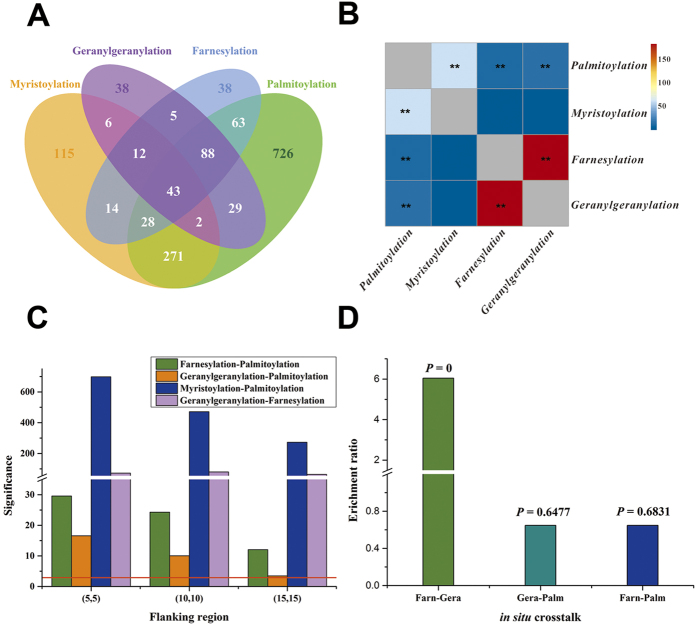
The co-regulatory mechanism of lipid modifications. (**A**) The distribution of lipid modified proteins in our collected sequence library. (**B**) The correlation between all six combinations of dual-lipid modification. The color strength represents the significance level calculated from the chi-square test. Positions in gray were nonsense dual-lipid modifications. Positions marked with an asterisk are cases with significant correlations (i.e. *P* < 0.05 or Significance > 2.99), while positions with two asterisks represent very significant correlations (i.e. *P* < 0.01 or Significance > 4.61). (**C**) The position distribution of different dual-lipid modifications. Four significantly correlated dual-lipid modifications were tested using chi-square test. The horizontal axis represents three tested flanking regions, while the vertical axis represents the significance of whether two types of lipid modification sites are trend to locate adjacently. The red line denote the significance level with probability lower than 0.05. (**D**) The *in situ* crosstalk between prenylation and palmitoylation. The x-axis represents three pairs of potential *in situ* crosstalk, while y-axis represents the enrichment ratio. Palm, Gera and Farn refer to S-Palmitoylation, S-Geranylgeranylation and S-Farnesylation, respectively.

To conduct a deep analysis of the co-regulatory mechanisms in lipid modifications, we constructed a comprehensive lipid modification dataset that contains 1221 manually collected lipidiation sites, 1447 palmitolyation proteins from high-throughput screen and 2257 GPS-Lipid predicted lipidiation sites. Interestingly, a large proportion of proteins were modified by at least two types of lipid groups, indicating a strong co-regulation was existed between different lipid modifications (Fig. 3A). It should be noted that the majority of the co-regulated proteins contain palmitoylation site, demonstrating that palmitoylation is a key modification that coordinate with other lipidation to modulate a diverse cellular functionality.

It is reported that proteins were often modified sequentially with different lipids. To explore how the different lipid modifications co-regulate, we performed a statistical analysis on the co-occurrence of all six combinations of dual-lipid modification. We found S-palmitoylation was significantly (*P* < 0.05) co-located with N-myristoylation, S-farnesylation and S-geranylgeranylation (Fig. 3B, Table S7). An obvious dual-lipid modification of S-farnesylation and S-geranylgeranylation were also detected (Fig. 3B).

We next analyzed the spatial relationships of different lipid-modified residues. As expected, we observed that S-palmitoylation sites are significantly (*P* < 0.05) enriched in proximal regions of N-myristoylation, S-farnesylation and S-geranylgeranylation sites (Fig. 3C, Table S8). Also, a highest spatial correlation was observed between S-farnesylation and S-geranylgeranylation (Fig. 3C). When extending the flanking region from (5,5) to (15,15), the level of significance was obviously decreased in all cases of dual-lipid modification. In this regard, we propose that the adjacent modification of two lipid groups are a key mechanism for dual-lipid modification, and palmitoylation should be the most essential lipid modification which required for the attachments of other lipid groups.

Since the two different types of prenylation will recognize the same C-terminal CAAX box, we speculated that the strong spatial relationship observed between S-farnesylation and S-geranylgeranylation was mainly caused by their *in situ* crosstalk. We performed a hypergeometric test to test whether S-farnesylation and S-geranylgeranylation will modify the same terminal cysteine. As palmitoylation can also occur in cysteine, we also calculated the significance of *in situ* crosstalk between palmitoylation and prenylation. Interestingly, we observed that S-farnesylation was significantly (*P* = *0*) *in situ* crosstalk with S-geranylgeranylation (Fig. 3D, Table S9). On the contrary, palmitoylation and prenylation was found to be unlikely to occur at the same cysteine residue. This result suggested that farnesylation and geranylgeranylation are two modifications with similar nature and functionalities. There may be a dynamic regulatory process that maintain the equilibrium between S-farnesylation and S-geranylgeranylation in mammalian cells, which is still needed further experimental verification.

## Discussion

Attachment of lipophilic groups is a widespread modification that has essential functions in eukaryotic cell. Over the past decades, at least four different types of lipid modifications are increasingly studied, which resulted numerous data. We collected all experimentally validated lipid modification data, based on which GPS-Lipid was developed to predict different types of lipid modification sites at the same time. Together with the comprehensive lipid modification dataset and visualization tools, GPS-Lipid is able to greatly assist the investigation of lipid modification for the research community. One of the most important function of GPS-Lipid is the ability to simultaneously identify different types of lipid modification, which would be of great help for the research of co-regulation between different types of lipid modification. As previously reported^2^,^37^, proteins were often attached sequentially with different types of lipid. Signal transducing proteins, such as guanine-nucleotide-binding protein-α (Gα) subunits and non-receptor tyrosine kinases, were first post-transnationally modified by palmityl group at N-terminal Cys residues. Following this, the adjacent Gly residue will be N-myristoylated and targeted to plasma membrane. Similar dual-lipid modifications were also observed in Ras proteins and other monomeric GTPases. At their C termini, those proteins were first modified by farnesyl group at the CAAX box, then, the adjacent palmitoylation will lead to a subcellular trafficking from endoplasmic reticulum to Golgi apparatus and plasma membrane. In consideration of these important physiological functions, the study of co-regulatory mechanisms between different lipid modifications will be distinctly important for further diagnostic and therapeutic considerations.

With GPS-Lipid, one can predict all potential lipid modification sites for a given interesting protein sequences, which provides specific targets for the subsequent experiments. We applied GPS-Lipid to our collected sequence library to systematically investigate the co-regulatory in lipidation. We observed that the four different types of lipidation are obviously crosstalk with each other. Specifically, a significant *in situ* crosstalk was detected between S-farnesylation and S-geranylgeranylation. However, as the training data set contained a limited amount of prenylation sites, the resolution of the predictions for S-farnesylation and S-geranylgeranylation will be insufficient. Therefore, the level of *in situ* crosstalk between S-farnesylation and S-geranylgeranylation may probably be overrepresented. By incorporating a larger data set for prenylation, it will be possible to further refine the prediction accuracy.

Although the current performance of GPS-Lipid is satisfactory, some lipid modification sites still cannot be properly predicted. Using our training dataset, we predicted the potential lipid modification sites with a default threshold and counted the number of mis-classified sites (Fig. S3). The statistical results suggest that our models are highly sensitive, however, there are still some negative sites incorrectly predicted as positive sites. To reveal the sequence characters of those false positive sites, we plot their motif representation using WebLogo^38^. Just as we have expected, the mis-classified lipid modification sites all share a very low sequence similarity and no apparent motif is detected (Fig. S4). Since the GPS algorithm is predicted based on groups, this kind of low similarity sites will be predicted in the non-consensus models. However, the dataset being assigned to non-consensus groups is very limited, therefore some false positive predictions will be introduced into our tool. An effective way to solve this problem is by gathering more low-similarity sites in the training dataset, in other words, to enlarge the training set in non-consensus groups.

In the future, further development of GPS-Lipid will be performed, which includes extension to other types of lipid modification, such as GPI-anchor addition, S-diacylglycerol addition and Cholesterol addition. Besides, a more precise GPS algorithm will be adopted in the next version of GPS-Lipid.

## Methods

### Data collection and preparation

The training data set in GPS-Lipid was manually collected by searching the scientific literatures (published before Nov. 2014) in the PubMed with keywords such as “Palmitoylation”, “Myristoylation”, “Farnesylation” and “Geranylgeranylation”. Here, we totally collected 737 S-palmitoylation sites in 361 proteins, 106 S-farnesylation sites in 97 proteins, 95 S- geranylgeranylation sites in 70 proteins and 283 N-myristoylation sites in 281 proteins. To provide full access to the above collected data set, an online database was then developed and the intact annotations from UniProt and NCBI were integrated. As previously described, to avoid any overestimation of prediction accuracy, the redundant sites should be removed, and the CD-HIT^39^ with a threshold of 40% sequence identity was used to single out homologous proteins. If two proteins are modified by lipid groups at the same position and present more than 40% sequence identity, only one protein was preserved. In particular, 65 palmitoylation sites was randomly selected from the non-redundant dataset to construct an additional test set. Due to data limitation, the additional test set for other lipid modifications were not constructed. For the preparation of training data sets, we took known lipid modification sites as the positive dataset, while all other non-modified residues, *i.e*. cysteine and glycine, in the same substrates were taken as the negative dataset. As a result, 579 S-palmitoylation sites, 226 N-myristoylation sites, 82 S-farnesylation sites and 71 S-geranylgeranylation sites were retained from 277, 226, 78 and 52 protein substrates as the final positive training data set (Supplementary table S3 – S6). While the corresponding negative dataset contains 3002 non-palmitoylated sites, 6754 non-myristoylated sites, 613 non-farnesylated sites and 192 non-geranylgeranylated sites.

To include as much as possible lipid modification sites, another 1259 high-throughput experimentally verified palmitoylated proteins was collected from PubMed. By using GPS-Lipid with a high threshold, the exact palmitoylation sites for those high throughput verified proteins were predicted and integrated into the lipid modification database. Notably, we also constructed a sequence library for further identifying the co-regulation mechanisms of lipid modifications by integrating the collected data set and high-throughput data set.

### The Algorithm of GPS-Lipid

Based on the hypothesis that similar short peptides exhibit similar biochemical properties and biological functions^31^, a previously described GPS algorithm^32^ was applied to predict potential lipid modification sites. In GPS algorithm, four sequential training procedures including modification sites clustering, Motif length selection (MLS), Weight training (WT) and Matrix mutation (MaM) were adopted for improving the prediction performance.

Before the training steps, we first took known lipid modification sites as the positive dataset, while all other non-modified residues (cysteine for palmitoylation and prenylation, glycine for myristoylation) in the same substrates were taken as the negative dataset. A detail statistics on training dataset was shown in Table S1. To enable the subsequent training steps, we extract the lipid modification site peptides as a lipid modified residue, *i.e*. cysteine and glycine, flanked by 30 residues upstream and 30 residues downstream.

In consideration of the fact that one type of post-translational modification (PTM) is capable of recognizing multiple motifs, the modification sites clustering approach was carried out to classify the lipid modification sites into several different groups based on recognition motifs. For N-myristoylation, the modified sites that follow an N-terminal MGXXXS/T motif were classified into a consensus set, while the other modified sites were classified into a non-consensus set. Similar strategy was also applied to prenylation. The farnesylation sites were classified into consensus and non-consensus class based on C-terminal CAAX motif. As for S-geranylgeranylation sites, the CAAX and CXC/CC motif were used. Since no apparent motifs were reported in S-palmitoylation, the palmitoylation sites were just clustered into groups using a previously described k-means approach. As a result, based on the sequence similarity of training data set, the S-palmitoylation sites were clustered into three optimal classes. In the k-means methods, we first calculated the similarity score between two given S-palmitoylation peptides using Equation 1.


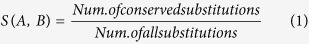


A conserved substitution is a substitution with a *Score*(*a, b*) *>* *0* in the BLOSUM62 matrix. The *S*(*A, B*) ranges from 0 to 1. The distance between the two *PSP*(*m, n*) is then defined as: *D*(*A,B*) = *1/S*(*A,B*). If *S*(*A,B*) = *0*, we simply let *D*(*A,B*) = *∞*. The *k*-means algorithm clusters the palmitoylation sites by exhaustive testing. First of all, two palmitoylation sites were randomly chosen as the centroids. Secondly, other positive sites were compared with the two centroids and the distances were calculated. With the shortest distance, the positive sites were then clustered into the corresponding groups. Thirdly, the centroids were updated with the highest average identity score. Optimal cluster can be obtained by iterative repeat of the second and third steps.

To evaluate the amino acid preference of modification enzymes, the WT method was adopted to optimize the scoring weight at each position of a lipid modification peptide. In WT process, the scoring strategy was defined as Equation 2.


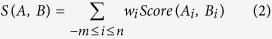


The *w*_*i*_ refers to the scoring weight of each position. Before the optimization steps, we re-illustrated the weight training process as Equation 3.


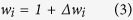


The *Δw*_*i*_ represents the numeric changes of scoring weight after training process. And hence, the weight training process is aimed at finding a set of *Δw*_*i*_ that can get an optimal performance. In GPS-Lipid, this step maximizes the Sn value of the LOO validation under a Sp of 90%. Furthermore, to improve the prediction robustness, a MaM process was subsequently performed. Similar to WT, the MaM process can also be described as Equation 4.


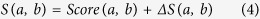


Where *S*(*a,b*) is the optimal substitute score for amino acid *a* and *b* with respect to lipid modification. *Score*(*a,b*) is the substitute score in BLOSUM62 matrix. *ΔS*(*a,b*) represents the numeric changes of substitute score for amino acid *a* and *b*. Thus, the MaM approach seeks for a set of *ΔS*(*a,b*) that maximize the prediction performance. In the most recent version of GPS algorithm^32^, the WT and MaM was performed by the Particle Swarm Optimization (PSO) strategy. Although the original PSO algorithm had exhibited a fast-converging behavior in our previous test^32^, we still found that, like other population-based optimization techniques, a guided particle located at a local optimum may have a risk of trapping the whole swarm and leading them to a premature convergence. To improve the performance of PSO, a number of PSO variants have been developed. Recently, inspired by the phenomenon of aging observed in nature, a variant of PSO, namely the PSO with an aging leader and challengers (ALC-PSO) was proposed by Weineng Chen^30^. According to Weineng Chen’s implementation, the ALC-PSO was integrated into GPS-Lipid. Using ALC-PSO, the problem of premature convergence in WT and MaM was overcome and the fast-converging feature of PSO was fully preserved. For more detail of the GPS algorithm, see also the supplementary methods and Fig. S1.

### Performance evaluation and comparison

To evaluate the performance of GPS-Lipid, the leave-one-out (LOO) and 4-, 6-, 8-, 10-fold cross validation was carried out on the training data set. In each validation, the sensitivity (*Sn*), specificity (*Sp*), accuracy (*Ac*), Mathew correlation coefficient (*MCC*) and precision (*Pr*) were calculated. Based on the above evaluation, the ROC curves were plotted and the areas under ROC (AUC) were computed. Particularly, an additional test set containing 65 S-palmitoylation sites was adopted to perform an extra evaluation on palmitoylation prediction. To further demonstrate the superiority of GPS-Lipid, we compared it with other existing tools. Since the Myristoylator did not predict precise myristoylation sites in protein substrates and the PrePS did not support batch prediction, the comparison will only perform among GPS-Lipid, CSS-Palm, CKSAAP-Palm and NMT. To avoid any bias, the same training data set used in the GPS-Lipid was adopted in other prediction tools. The prediction results along with the predicted scores are collected from the above three pieces of software. To draw the ROC curves, we change the prediction cutoff according to the collected scores and the performances under different cutoff are calculated. Specifically, when performing the comparison, the prediction threshold for CSS-Palm was set as ‘all’. While for CKSAAP-Palm, we set the penalty factor *C* and *Gamma* as 100 and 0.0000015, respectively. In NMT, a default parameter was used.

### Statistical analysis of the co-regulatory mechanisms between different lipid modifications

Lipid modifications are known as key biological processes that impact diverse cellular functionality by modulating the membrane targeting, subcellular trafficking, intracellular sorting and stability of proteins in most eukaryotes. However, due to the unavailability of comprehensive bioinformatics resources, the co-regulatory mechanisms were seldom performed. In order to analyze the relationship between different lipid modification sites, we designed the following statistical methods. Firstly, we extracted all our collected lipid-modified proteins from the original dataset and constructed a sequence library containing 1479 proteins. Then, the GPS-Lipid was applied to predict potential lipid modification sites with a high threshold. All the predicted sites were subsequently mapped to the experimentally verified sites and the reduplicative sites were removed. Totally, there are 3729 amino acid residues annotated as modifying by lipid groups.

As reported in literatures, dual-lipid modifications are important co-regulatory mechanisms that orchestrate a wide range of molecular processes in eukaryotic cell. With a chi-square test, we statistically examined the dependency for all six combinations of dual-lipid modifications in our collected sequence library. Here, we take the case of palmitoylation-myristoylation dual-lipid modification as an example to describe the statistical method. Before the statistics, a statistical hypothesis is made as follow: the presence of a myristoylation sites in a protein is not relevant to the palmitoylation sites in the same protein substrate. Based on this hypothesis, the number of proteins that contain both myristoylation sites and palmitoylation sites is calculated. Similarly, the number of proteins that just contain myristoylation sites or just contain palmitoylation sites are also calculated. Then, the probability is obtained from a chi-square distribution.

After identifying significant dual-lipid modifications, we next further investigate the spatial relationship between different lipid modifications. Based on the prediction result from GPS-Lipid, we looked through the flanking regions, such as (5,5), (10,10) and (15,15), around a given lipid modification site and analyzed whether another type of lipid modification would be significantly occurred in those proximal regions with a chi-square test. Again, we took the palmitoylation-myristoylation dual-lipid modification as an example to describe the statistical method. Firstly, for all predicted myristoylated glycine residues, we counted the number of myristoylation sites with at least one palmitoylation sites in flanking regions. Also, the number of myristoylation sites that without any palmitoylation sites in flanking regions is counted. Accordingly, two similar statistics were also performed on other non-myristoylated glycine residues. With a chi-square distribution, the probability is calculated.

At last, since the prenylation and palmitoylation are both modified the cysteine, a hypergeometric test was carried out to evaluate the *in situ* crosstalk between prenylation and palmitoylation. In this analysis, we tested whether prenylation is more inclined to be happened on palmitoylated cysteines. A more detail description on the statistical methods are presented in supplementary methods.

## Additional Information

**How to cite this article**: Xie, Y. *et al*. GPS-Lipid: a robust tool for the prediction of multiple lipid modification sites. *Sci. Rep*. **6**, 28249; doi: 10.1038/srep28249 (2016).

## Supplementary Material

Supplementary Information

Supplementary Table S4

Supplementary Table S7
