# Supplementary material for: GPS-Lipid: a robust tool for the prediction of multiple lipid modification sites
Source: Sci Rep. 2016 Jun 16;6:28249. doi: 10.1038/srep28249 (PMC4910163; doi:10.1038/srep28249)

**GPS-Lipid: a robust tool for the prediction of multiple lipid modification sites**

**Yubin Xie^1, †^, Yueyuan Zheng^1, †^, Hongyu Li^1, †^, Xiaotong Luo^1^, Zhihao He^1^, Shuo Cao^1^, Yi Shi^1^, Qi Zhao^1,2,3^, Yu Xue^4^, Zhixiang Zuo^1,2^ and Jian Ren^1,2,3,*^**

^1^State Key Laboratory of Biocontrol, School of Life Sciences, Sun Yat-sen University, Guangzhou, Guangdong 510275, China

^2^Sun Yat-sen University Cancer Center, State Key Laboratory of Oncology in South China, Collaborative Innovation Center for Cancer Medicine, Guangzhou 510060, China

^3^Collaborative Innovation Center of High Performance Computing, National University of Defense Technology, Changsha 410073, China

^4^Department of Biomedical Engineering, College of Life Science and Technology, Huazhong University of Science and Technology, Wuhan 430074, China

*To whom correspondence should be addressed.

Tel/Fax:+86 20 39943788; Email: [renjian.sysu@gmail.com](mailto:renjian.sysu@gmail.com).

†The authors wish it to be known that, in their opinion, the first three authors should be regarded as joint First Authors.

# SUPPLEMENTARY METHODS

## The fourth generation GPS algorithm

In GPS-Lipid, our GPS series algorithms were greatly improved and updated into the GPS 4.0 algorithm. Based on the hypothesis that similar short peptides can exhibit similar biochemical properties and biological functions[^1^](#_ENREF_1), a lipid modification site peptide *LPSP(m, n)* is defined as a lipid modified residue, *i.e.* cysteine and glycine, flanked by m residues upstream and n residues downstream. As previously described[^2^](#_ENREF_2), the scoring strategy was not modified and directly applied to calculate the similarity score between two *LPSP(m, n)* items using Equation (1):

$\text{S(A,B)}=\sum_{-\text{m}\leq\text{i}\leq\text{n}} \text{Score(A}_{\text{i}}\text{,B}_{\text{i}}\text{)}$ (1)

The *Score(A_i_, B_i_)* represents the substitute score of two residues *A_i_* and *B_i_* in an amino acids substitution matrix, *e.g.*, BLOSUM62 matrix. The *S(A,B)* value is redefined as zero when *S(A,B) < 0*. When all *S (A,B)* are given, the GPS algorithm calculates the average score between the peptide and the experimentally verified lipid modified peptides. If the average score is larger than a preset threshold, the corresponding site is predicted as a lipid modification site. Previously, the GPS 3.0 algorithm adopted four sequential procedures including k-means clustering, Motif Length Selection (MLS), Weight Training (WT) and Matrix Mutation (MaM), for improving the prediction performance. For a better accuracy and faster training process, the four steps of performance improving approaches were refined as Motif clustering, MLS, WT with Particle Swarm Optimization using an aging leader and challengers strategy (ALC-PSO) [^3^](#_ENREF_3), and MaM with ALC-PSO. The detailed algorithms were described as below:

1. **Motif clustering.** Based on the fact that one type of post-translational modification (PTM) is capable of recognizing multiple motifs, the *LPSP(m,n)* items from the training data set were classified into several different groups based on recognition motifs. For N-Myristoylation, we clustered the peptides into a consensus group and non-consensus group based on the N-terminal MGXXXS/T motif. While for farnesylation, the peptides were classified into C-terminal CAAX group and non-consensus group. Since the geranylgeranyltransferase can recognized a distinct C-terminal CXC/CC motif in Rab protein family, the geranygeranylated peptides were clustered into a CAAX group, CXC/CC group and a non-consensus group. Due to the lack of apparent recognition motif, palmitoylation peptides were just clustered into groups using a k-means approach. According to the data size, the parameter k was set to 3, that is, clustering into three groups.
2. **Motif Length Selection (MLS).** This step determines the optimal motif length for each *LPSP(m, n)* cluster. With an extensively test, the LOO validation for all combination of *LPSP(m, n)* (*m* = 1…30, *n* = 1…30) were carried out. In this study, the *Sp* was fixed at 90% to determine the optimal *LPSP(m, n)* with the highest *Sn* value.
3. **Weight Training (WT) with ALC-PSO algorithm.** To evaluate the amino acid preference of modification enzyme, the weight training method was adopted to optimize the scoring weight at each position of *LPSP(m,n)*. In our previous work, the WT and MaM procedures were implemented in an original PSO algorithm, which will have a risk of trapping the whole swarm and leading them to a premature convergence. Thus, in this new generation GPS algorithm, the ALC-PSO method was integrated. In WT process, the scoring strategy was redefined as Equation (2).

$\text{S(A,B)}=\sum_{-\text{m}\leq\text{i}\leq\text{n}} \text{w}_{\text{i}}\text{Score(A}_{\text{i}}\text{,B}_{\text{i}}\text{)}$ (2)

The *w_i_* refers to the scoring weight of each position. Before the optimization steps, we re-illustrated the weight training process as Equation (3).

$\text{w}_{\text{i}}=1+\text{Δw}_{\text{i}}$ (3)

The *Δw_i_* represents the numeric changes of scoring weight after training process. And hence, the weight training process is aimed at finding a set of *Δw_i_* that can get an optimal performance. In GPS-Lipid, this step maximizes the *Sn* value of the LOO validation under a *Sp* of 90%.

1. **Matrix mutation (MaM) with ALC-PSO algorithm.** It was previously shown that the Matrix Mutation approach can efficiently enhance the prediction performance of GPS predictor[^1^](#_ENREF_1). Therefore, the MaM process was adopted in this work. Similarly, the MaM process can also be described as Equation (4).

$\text{S(a,b)}=\text{Score(a,b)}+\text{ΔS(a,b)}$ (4)

Where *S(a,b)* is the optimal substitute score for amino acid *a* and *b* with respect to palmitoylation. *Score(a,b)* is the substitute score in BLOSUM62 matrix. *ΔS(a,b)* represents the numeric changes of substitute score for amino acid *a* and *b*. Thus, the MaM approach seeks for a set of *ΔS(a,b)* that maximize the prediction performance. The LOO validation was used as a fitness function in ALC-PSO. During the training step, the *Sp* value was fixed at 90%.

The remarkable difference between original PSO and ALC-PSO is that the leader of the swarm in ALC-PSO will age with a limited lifespan. During the optimization steps, the lifespan of a leader is adjusted adequately according to its leading power. When the leader reach its end-of-life, a new particle will generate and challenge the leader. Therefore, with this aging mechanism, the misleading effects caused by the unchanged leader should be avoided. The main processes of ALC-PSO is briefly illustrated in Figure S1 and the detail description is given as follow.

**Step 1. Initialization.** The ALC-PSO initializes a population array of particles with random current position and velocities on D-dimensions. In this case, the randomly generated Δwi and ΔS(a,b) were directly assigned to the current position. For each particle, the LOO validation is used to evaluate the prediction performance in the current position and the particle with the best performance is selected as a leader. The age of the leader is initialized as *θ = 0* and the lifespan *Θ* is set to an initial value *Θ_0_*.

**Step 2. Update of velocity and position.** The velocity and position of each particle in the swarm is updated according to Equation (5).

$\left\{\begin{matrix} \text{v}_{\text{i}}=\text{ω}\times\text{v}_{\text{i}}+\text{Rand(0,}\phi_{1}\text{)}\otimes\text{(p}_{\text{i}}-\text{x}_{\text{i}}\text{)}+\text{Rand(0,}\phi_{2}\text{)}\otimes\text{(p}_{\text{g}}-\text{x}_{\text{i}}\text{)} \\ \text{x}_{\text{i}}=\text{x}_{\text{i}}+\text{v}_{\text{i}}\begin{matrix} \begin{matrix} & & & & \end{matrix} & & & \begin{matrix} \begin{matrix} & & & & \end{matrix} & & & & \end{matrix} \end{matrix} \end{matrix}\right.$ (5)

Where *ω* represents as inertia weight. *Rand(0, φ_1_)* and *Rand(0, φ_2_)* is two random functions generated real numbers distributed in [0, *φ_1_*] and [0, *φ_2_*]. is component-wise multiplication. Notably, the range of each *v_i_* is limited from *–V_max_* to *V_max_*.

**Step 3. Update of pBest and Leader.** In each particle *i*, we compared the current position to the previously observed best position. If the current position is better than previously best position, it will become the new *pBest_i_*. In addition, if the best position found in this iteration is better than the leader, then the leader is replaced by the best position in this iteration.

**Step 4. Lifespan control.** This is the most important step in ALC-PSO. After updating the leader, the leading power is evaluated in a lifespan controller. Then, the lifespan of the current leader is adjusted based on its leading power. Also, the age *θ* of the leader is increased by 1. Next, the lifespan controller will check whether the lifespan of the leader is exhausted. If the *θ ≥ Θ*, the algorithm will go to step 5. Otherwise, it will go to step 7.

**Step 5. Generating new challenger.** When the lifespan of the leader is exhausted, a new particle is generated and used to challenge the leader.

**Step 6. Evaluating the new challenger.** After generating of new challenger, the leading power of the challenger is evaluated. If the challenger has enough leading power, it will replace the old leader and becomes a new leader. Otherwise, the old leader will preserve and continue to lead the whole swarm.

**Step 7. Checking of the stopping criteria.** If the performance of LOO validation is no longer improved, the algorithm will terminate. However, if the swarm still have a probability of obtaining a better performance, the algorithm will go to step 2 for a new round of iteration.

## The chi-square test on all six combinations of dual-lipid modifications

The dual-lipid modifications are important co-regulatory mechanisms that orchestrate a wide range of molecular processes in eukaryotic cell. To evaluate the significance of different dual-lipid modification in our collected sequence library, we performed the following statistical test.

Firstly, we used GPS-Lipid with a high threshold to predict the potential lipid modification sites in the collected sequence library. Then, the predicted sites were combined with the experimentally verified sites and the redundant sites were removed. Totally, 3792 lipid modification sites were predicted from 1479 protein sequences. Of which, 2634 cysteine residues were annotated as S-palmitoylation sites, 541 glycine residues were annotated as N-myristoylation sites, 347 cysteine residues were annotated as S-farnesylation sites and 270 cysteine residues were annotated as S-geranylgeranylation sites. Based on this data set, we examined the following pairs of dual-lipid modification: palmitoylation-myristoylation, palmitoylation-farnesylation, palmitoylation-geranylgeranylation, myristoylation-farnesylation, myristoylation-geranylgeranylation, farnesylation-geranylgeranylation.

Here, we take the case of palmitoylation-myristoylation dual-lipid modification as an example to descript the statistical method. Before the statistics, a statistical hypothesis is made as follow: the presence of a myristoylation sites in a protein is not relevant to the palmitoylation sites in the same protein substrate. Based on this hypothesis, the number of proteins that contain both myristoylation sites and palmitoylation sites is calculated. Similarly, the number of proteins that just contain myristoylation sites or just contain palmitoylation sites are also calculated. With a chi-square test, the probability of the relevance between palmitoylation and myristoylation is computed. If probability is lower than 0.05, we refused the null hypothesis, that is, a strong dependency is observed between palmitoylation and myristoylation. Conversely, if the probability is larger than 0.05, dependency is not observed in the tested case. The other dual-lipid modifications are tested using the same procedure. When the calculation is finished, we transformed the probability to significance using Equation (6) and plotted the significance in a heat map.

$\text{Sig.}=-\text{ln(P)}$ (6)

Where *Sig.* denote the significance, *P* denote the probability.

## The chi-square test on the spatial relationship between different lipid modification sites

After the identification of significant dual-lipid modifications, we next analyze the spatial relationship between two types of lipid modification sites. Based on the predicted lipid modification sites, we looked through the flanking regions such as (5, 5), (10, 10) and (15, 15) with at least one lipid modification sites around another types of lipid modification site. Again, we took the palmitoylation-myristoylation dual-lipid modification as an example to descript the statistical method. Firstly, for all predicted myristoylated glycine residues, we counted the number of myristoylation sites with at least one palmitoylation sites in flanking regions. Also, the number of myristoylation sites that without any palmitoylation sites in flanking regions is counted. Accordingly, two similar statistics were also performed on other non-myristoylated glycine residues. With a chi-square distribution, we tested the hypothesis that whether a myristoylation site is located adjacently to a palmitoylation site. When the calculation is finished, we transformed the probability to significance using Equation (6) and plotted the significance in a bar chart.

## The hypergeometric test on the *in situ* crosstalk between different lipid modifications

Since the prenylation and palmitoylation are both modified the cysteine, a hypergeometric test on the *in situ* crosstalk between prenylation and palmitoylation were carried out. As an example, here we illustrate the statistical procedure of palmitoylation and farnesylation. In the analysis, we computed the following statistics.

- The number of cysteines in full-length sequence. (*N*)
- The number of palmitoylated cysteins in full-length sequence. (*n*)
- The number of geranylgeranylated cysteins in full-length sequence. (*M*)
- The number of geranylgeranylated cysteins that simultaneously modified by palmityl. (*k*)

Given the above statistics, we calculated the enrichment ratio (ER) of geranylgeranylation that *in situ* crosstalk with palmitoylation using Equation (7).

$\text{ER }=\text{ }\frac{\text{k/m}}{\text{n/N}}$ (7)

With a hypergeometric distribution, the probability were also calculated as shown in Equation (8).

(8)

## The evaluation of GPS-Lipid for its time and memory consumption

Using the training dataset for those four types of lipid modifications, we tested the time and memory consumption for GPS-Lipid, CKSAAP-Palm and NMT. A detail comparison results were shown in Table S2. Obviously, GPS-Lipid is the best in efficiency among the three pieces of software. For memory consumption, the CKSAAP-Palm hog up the least memory. Since GPS-Lipid is a prediction tool with graphical interface, this feature will incurs overhead of extra memory. But for this memory consumption, GPS-Lipid can surely be supported by most of the mainstream PCs. Specifically, the memory consumption for NMT cannot be validated because that NMT only provided an online version.

# References

1 Xue, Y. *et al.* GPS 2.0, a tool to predict kinase-specific phosphorylation sites in hierarchy. *Molecular & cellular proteomics : MCP* **7**, 1598-1608, doi:10.1074/mcp.M700574-MCP200 (2008).

2 Ren, J. *et al.* CSS-Palm 2.0: an updated software for palmitoylation sites prediction. *Protein engineering, design & selection : PEDS* **21**, 639-644, doi:10.1093/protein/gzn039 (2008).

3 Chen, W. N. *et al.* Particle Swarm Optimization With an Aging Leader and Challengers. *Evolutionary Computation, IEEE Transactions on* **17**, 241-258, doi:10.1109/TEVC.2011.2173577 (2013).

**SUPPLEMENTARY TABLES**

**Table S1 – A detail statistic on the training data set in GPS-Lipid.** a. The total number of positive sites. b. The total number of negative sites. c. Palm refers to S-Palmitoylation. Myri refers to N-Myristoylation. Farn refers to S-Farnesylation. Gera refers to S-Geranylgeranylatiion.

|  | Palm*^c^* | Myri*^c^* | Farn*^c^* | Gera*^c^* |
| --- | --- | --- | --- | --- |
| Pos*^a^* | 579 | 226 | 82 | 71 |
| Neg*^b^* | 3002 | 6754 | 613 | 192 |

**Table S2 – The comparison of time and memory consumption for three existing tools.**

| Software | Time consumption (seconds) | Memory consumption (Mb) |
| --- | --- | --- |
| GPS-Lipid (S-Palmitoylation) | 3.6 | 9.6 |
| CKSAAP-Palm | 45000 | 2.1 |
| GPS-Lipid (N-Myristoylation) | 0.6 | 9.6 |
| NMT | 21.5 | N/A |

**SUPPLEMENTARY FIGURES**

**Figure S1 – The flowchart of ALC-PSO.** This flowchart was modified from Weineng Chen’s paper [^3^](#_ENREF_3).


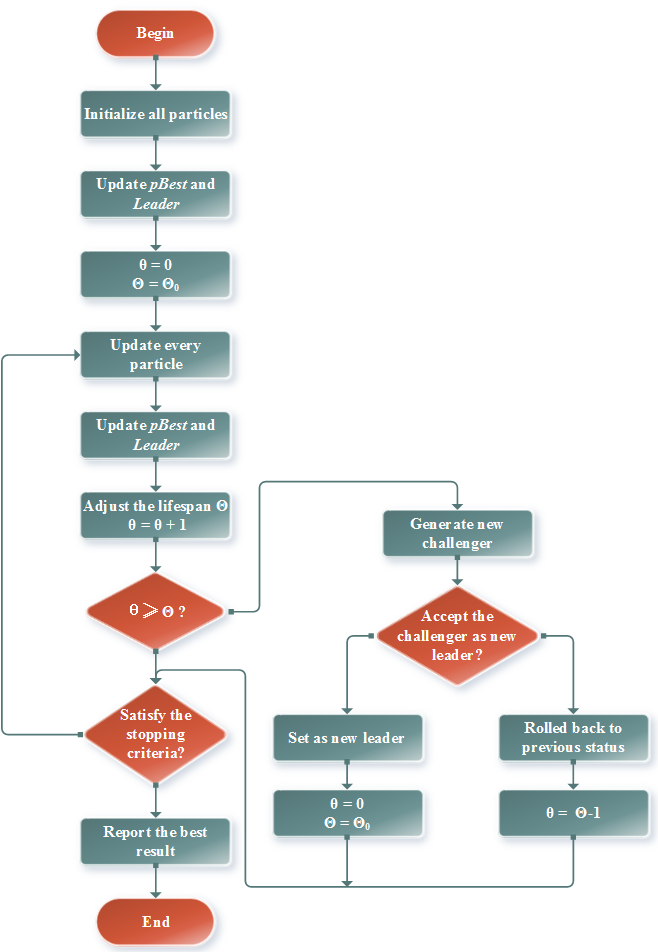


**Figure S2 – Precision-recall curves of GPS-Lipid.** (**A**) The precision-recall curve for S-palmitoylation. (**B**) The prediction-recall curve for N-myristoylation. (**C**) The prediction-recall curve for S-farnesylation. (**D**) The prediction-recall curve for S-geranylgeranylation.


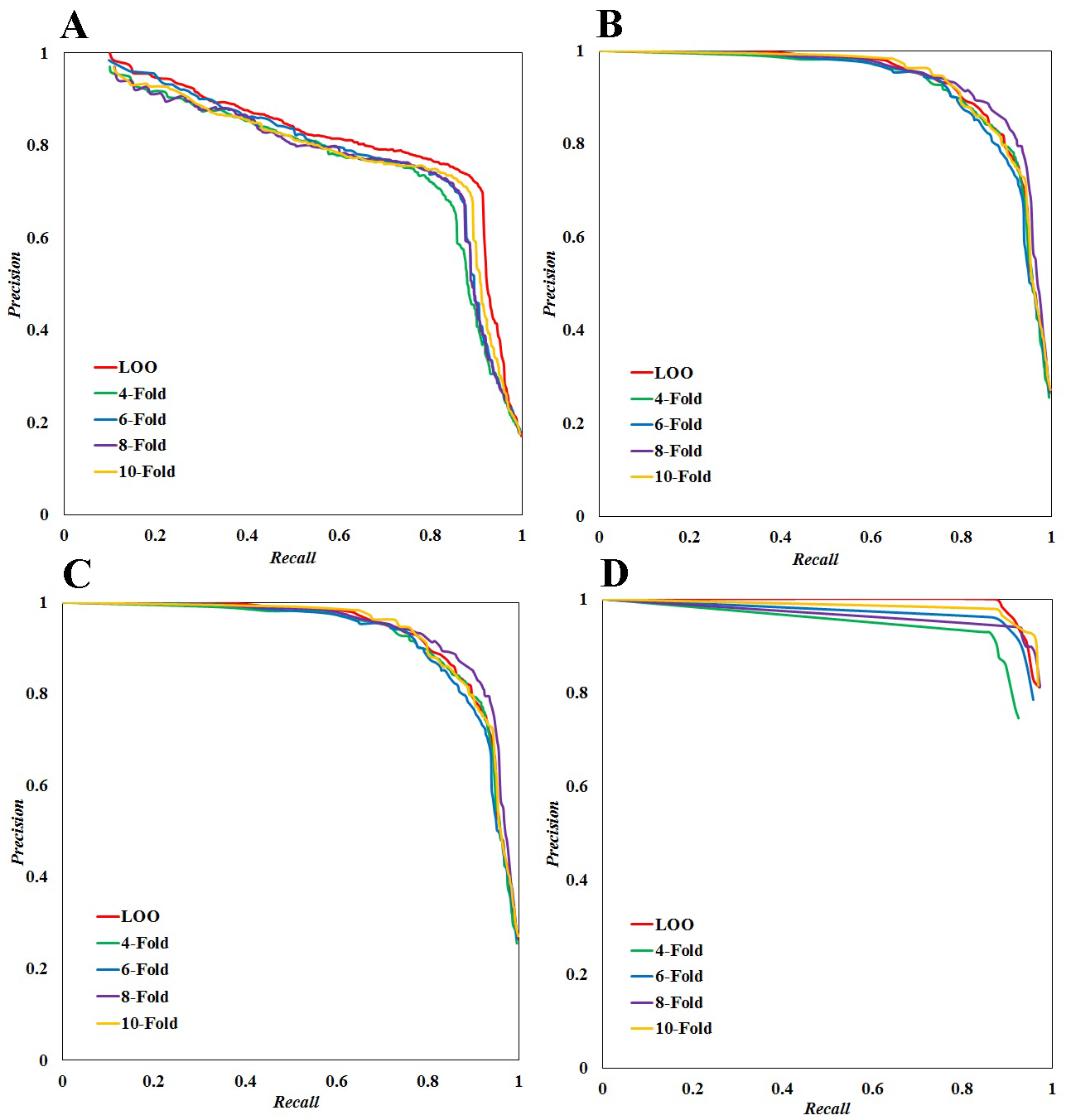


**Figure S3 – The distribution of mis-classified lipid modification sites.** The training dataset of palmitoylation, myristoylation and prenylation were used in GPS-Lipid. Then, we predicted the potential lipid modification sites under a default threshold. The Venn diagram was plotted to reveal the distribution of false positive and false negative sites.


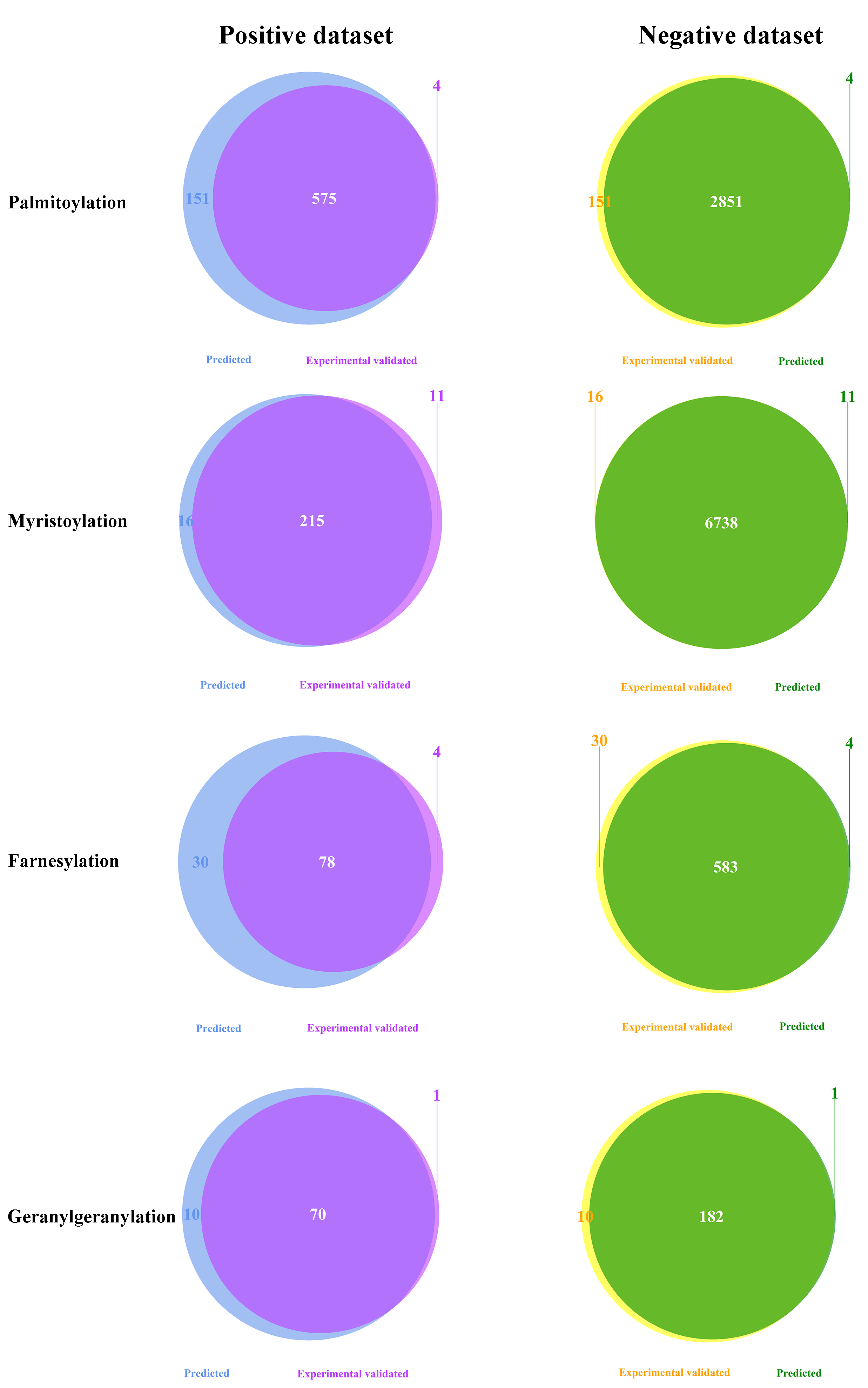


**Figure S4 – The motif in false positive sites.** We plotted the motif representation for the false positive sites of palmitoylation, myristoylation and prenylation in WebLogo. A window size of (7,7) is used to extract the lipid modification peptides and the information entropy was adopted to indicate the sequence conservation at each position.


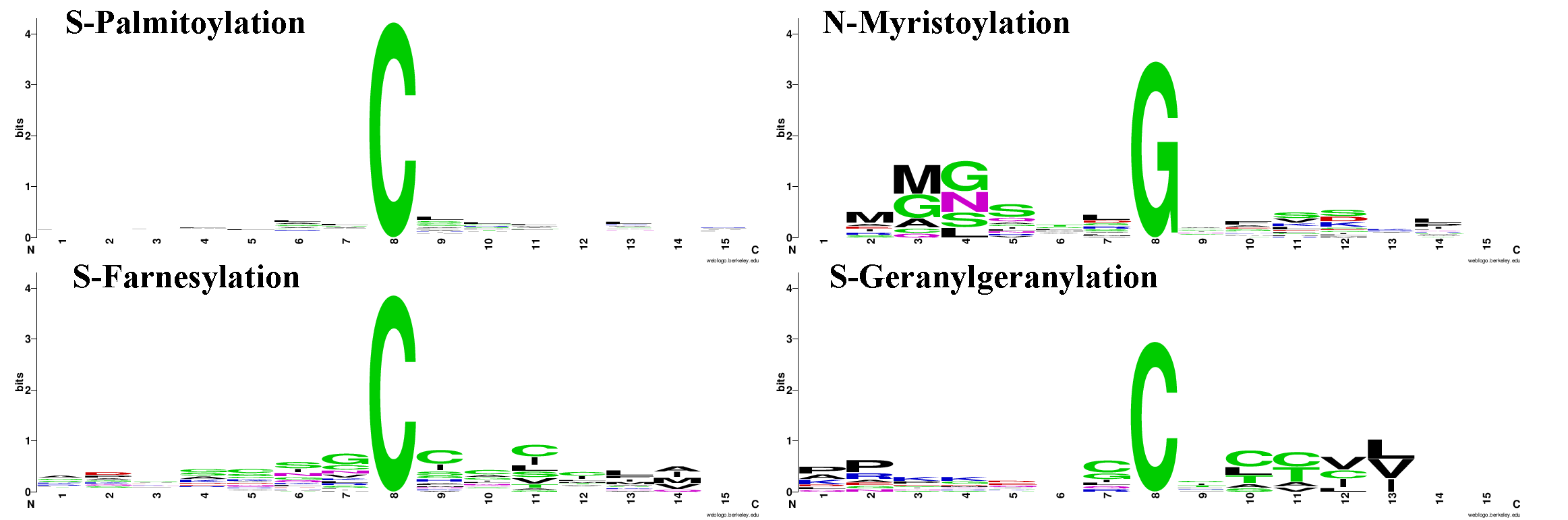

Supplement: Supplementary Information [file srep28249-s1.doc]
